# Development and Validation of a Prognostic Nomogram for Gastric Cancer Based on DNA Methylation-Driven Differentially Expressed Genes

**DOI:** 10.7150/ijbs.41587

**Published:** 2020-02-10

**Authors:** Yi Bai, Chunlian Wei, Yuxin Zhong, Yamin Zhang, Junyu Long, Shan Huang, Fucun Xie, Yantao Tian, Xi Wang, Haitao Zhao

**Affiliations:** 1 Department of Liver Surgery, Peking Union Medical College Hospital, Chinese Academy of Medical Sciences & Peking Union Medical College (CAMS & PUMC), Beijing, China.; 2 Department of Immunology, Beijing Key Laboratory for Cancer Invasion and Metastasis, Advanced Innovation Center for Human Brain Protection, School of Basic Medical Sciences, Capital Medical University, Beijing, China.; 3 Department of Pancreatic and Gastric Surgery, National Cancer Center/National Clinical Research Center for Cancer/Cancer Hospital, Chinese Academy of Medical Sciences and Peking Union Medical College, Beijing, China.; 4 Department of Hepatobiliary Surgery, First Central Hospital, Tianjin, China.

**Keywords:** nomogram, risk score, gastric cancer, DNA methylation, prognosis

## Abstract

**Background/Aims**: The incidence of gastric cancer (GC) ranks fifth among common tumors and GC is the third leading cause of cancer-related death worldwide. The aim of this study was to develop and validate a nomogram for predicting the overall survival (OS) of patients with GC.

**Methods**: DNA methylation (DNAm)-driven genes were identified by integrating DNAm and gene expression profiling analyses from The Cancer Genome Atlas (TCGA) GC cohort. Then, a risk score model was built based on Kaplan-Meier (K-M), least absolute shrinkage and selector operation (LASSO), and multivariate Cox regression analyses. After analyzing the clinical parameters, a nomogram was constructed and assessed. Another cohort (GSE62254) was used for external validation.

**Results**: Thirteen differentially expressed DNAm-driven genes were narrowed down to a six-gene signature (*PODN*, *NPY*, *MICU3*, *TUBB6* and *RHOJ* were hypermethylated, and *MYO1A* was hypomethylated), which was associated with OS (*P* < 0.05) after survival and LASSO regression analyses. These differentially expressed genes (DEGs) with altered DNAm statuses were included in the prognostic risk score model. The univariate Cox regression analysis indicated that risk score, age, and number of positive lymph nodes were significantly associated with survival time in GC patients. The multivariate Cox regression analysis also indicated that these variables were significant prognostic factors for GC. A nomogram including these variables was constructed, and its performance in predicting the 1-, 3- and 5-year survival outcomes of GC patients was estimated through time-dependent receiver operating characteristic (ROC) curves. In addition, the clinical benefit of this model was revealed by decision curve analysis (DCA). Pathway enrichment analysis suggested that these DNAm-driven genes might impact tumor progression by affecting signaling pathways such as the “ECM RECEPTOR INTERACTION” and “DNA REPLICATION” pathways.

**Conclusions**: The altered status of the DNAm-driven gene signature (*PODN*, *MYO1A*, *NPY*, *MICU3*, *TUBB6* and *RHOJ*) was significantly associated with the OS of GC patients. A nomogram incorporating risk score, age and number of positive lymph nodes can be conveniently used to facilitate the individualized prediction of OS in patients with GC.

## Introduction

The incidence and cancer-related death of gastric cancer (GC) rank fifth and third, respectively, among those of common tumors 1, 2. Curative surgery, chemotherapy that combines platinum with fluoropyrimidines or paclitaxel (PTX) plus ramucirumab 3-5 and target therapies remain the most common treatment options. Because GC is frequently diagnosed in advanced stages, the prognosis of GC is still not satisfactory 6. Aside from progress in treatment approaches, exploring efficient biomarkers for early identification is another important precaution to improve the prognosis of GC patients. Compared to traditional diagnostic methods, more specific and sensitive biomarkers demonstrate promising value in early diagnosis, predicting prognosis and even therapeutic responses.

In pursuit of predictive factors for patients with GC, an increasing number of studies have identified some valuable biomarkers, such as fibroblast growth factor receptor (FGFR) 7 and disrupted in renal cancer 1 (DIRC1) 8. However, prognostic biomarkers for GC are still limited, and due to a lack of specificity and sensitivity, few markers have been adopted for application.

DNA methylation (DNAm) is an important epigenetic event that can influence pretranscriptional gene silencing, genetic imprinting, X-chromosome inactivation (XCI), genome stability, and cell fate determination 9. De novo methyltransferases, namely*,* DNMT3A and DNMT3B 10, 11, play a vital role in tumorigenesis mainly by methylating CpG dinucleotides 12. Aberrant DNAm in the promoter regions is generally believed to be a hallmark of tumors, which often leads to the transcriptional silencing of tumor suppressor genes (TSGs) and the abnormal activation of oncogenes in tumor cells 13. There is evidence that abnormal DNAm frequently occurs in early-stage tumors 14, and these alterations are relatively stable and potentially reversible therapeutically 15-17. Hence, the deregulated DNAm status shows prospective utility as a biomarker for early diagnosis, prognosis and clinical decision-making for a variety of tumors.

Despite extensive studies on the relationship between abnormal DNAm and the prognosis of patients with GC, individualized prognostic models considering the DNAm-driven gene signature have rarely been reported. By integrating methylation and mRNA expression profile data, we identified prognosis-related differentially expressed genes (DEGs) with altered DNAm status and established a risk score model after Kaplan-Meier (K-M) and LASSO analyses. Finally, we established a nomogram via an integrated analysis of both the DNAm signature and clinicopathologic risk factors to predict overall survival (OS) in patients with GC, which was then validated in another Gene Expression Omnibus (GEO) cohort.

## Materials and Methods

### Patient Population and Clinical Data

All The Cancer Genome Atlas (TCGA) data are available through the NIH Genomic Data Commons (GDC). Here, TCGA level-3 molecular data and the corresponding clinical files were obtained from the GDC (2019/1/21 analysis archive). The methylation levels of genes were scored using β values ranging from 0 to 1 (unmethylated to totally methylated).

### Identification of DEGs between GC and Nontumorous Tissues

We identified the DEGs between 343 GC tissues and thirty adjacent nontumorous gastric tissues from the training dataset (HTSeq-Counts of TCGA-STAD transcriptome profiling with complete prognostic information and diagnosed as adenomas and adenocarcinomas) using the DESeq package 18. An absolute log2-fold change (|FC|) of > 1 and an adjusted *P* value of < 0.05 were set as cutoff criteria. Visualization of the six DNAm-driven gene expression patterns between GC and noncancer gastric tissues was performed with Prism 8.0 (GraphPad, San Diego, CA, USA).

### Approach used to Identify DNAm-Driven Genes

Gene expression data and DNAm data were integrated with the same TCGA barcode structure. DNAm-driven genes are those genes whose DNAm levels are negatively correlated with the mRNA expression level after linear regression analysis. Simultaneously, the differential DNAm state between GC tissues and adjacent nontumorous gastric tissues was compared by employing the Wilcoxon rank-sum test method as described previously 19.

### Functional Enrichment Analysis

Seventy-one DNAm-driven genes were subjected to Gene Ontology (GO) and pathway enrichment analyses, with the help of the Database for Annotation, Visualization and Integrated Discovery (DAVID) Bioinformatics Tool (version 6.8) and ConsensusPathDB (http://cpdb.molgen.mpg.de/), respectively.

### Feature Selection and Building the Predictive Signature

Initially, K-M analysis was utilized to evaluate the relationship between DNAm-driven genes and the survival time of GC patients. To further narrow the scope of the candidate DNAm-driven genes, we adopted the LASSO binary logistic regression model and multivariate Cox regression after primary filtration. The linear combination of the regression coefficient derived from the multivariate Cox regression model (β) multiplied by its mRNA level generated a prognostic risk score with six genes.

### Development and Validation of the Risk Score Model

Employing X-tile 20 to determine appropriate cut-off values, we separated patients into low- and high-risk groups, after which the K-M survival curves were plotted using the survival data of the two groups of GC patients. The potential of the predictive signature was assessed in the primary cohort and validated in the GSE62254 cohort.

### Screening of Prognostic Factors

The significance of the risk score model and other traditional clinical characteristics to predict OS in GC patients was evaluated by univariate Cox regression analysis. Then, confounding factors were excluded through multivariable logistic regression analysis. The statistical significance levels were all two-sided at 0.05, and the hazard ratio (HR) and its 95% confidence interval (CI) were also calculated.

### Development and Assessment of the Nomogram in the TCGA Dataset

Multivariate Cox regression analysis distinguished significant predictive factors, from which we built a predictive model. To evaluate the performance of the nomogram in the primary cohort, we assessed the calibration of OS probability at different years for patients with GC by applying the Hosmer-Lemeshow test to plot calibration curves.

Harrell's concordance index (C-index) was measured to quantify the discrimination performance of the nomogram. The nomogram was validated with 1000 bootstrap resamples to calculate a robust C-index. The value of the C-index ranged from 0.5 (indicates random chance) to 1.0 (indicates perfect capacity to correctly distinguish the outcome via this model).

We also conducted a time-dependent ROC analysis 21 to measure the predictive performance of the nomogram. Then, decision curve analysis (DCA) 22 was employed to quantify the clinical utility with clinical consequences of a decision considered.

### External Validation of the Nomogram

In the validation phase, we verified the nomogram in the GEO by using another GC cohort, GSE62254.

### Copy Number Variation (CNV), Mutation Characteristics and Gene Set Enrichment Analysis (GSEA) of Six DNAm-Driven Genes

Graphic illustrations of CNV and the six-gene mutation profiles in all GC tissues from the TCGA dataset were obtained from cBioPortal (http://www.cbioportal.org/). GSEA was performed using gsea-3.0.jar software according to the methods described in the user guide (http://software.broadinstitute.org/gsea/index.jsp).

### Statistical Analysis

All statistical analyses were conducted with R software (version 3.5.2). All statistical tests were two-sided, and *P* values less than 0.05 were considered statistically significant.

## Results

### Identification of DEGs in GC

**Figure 1:**
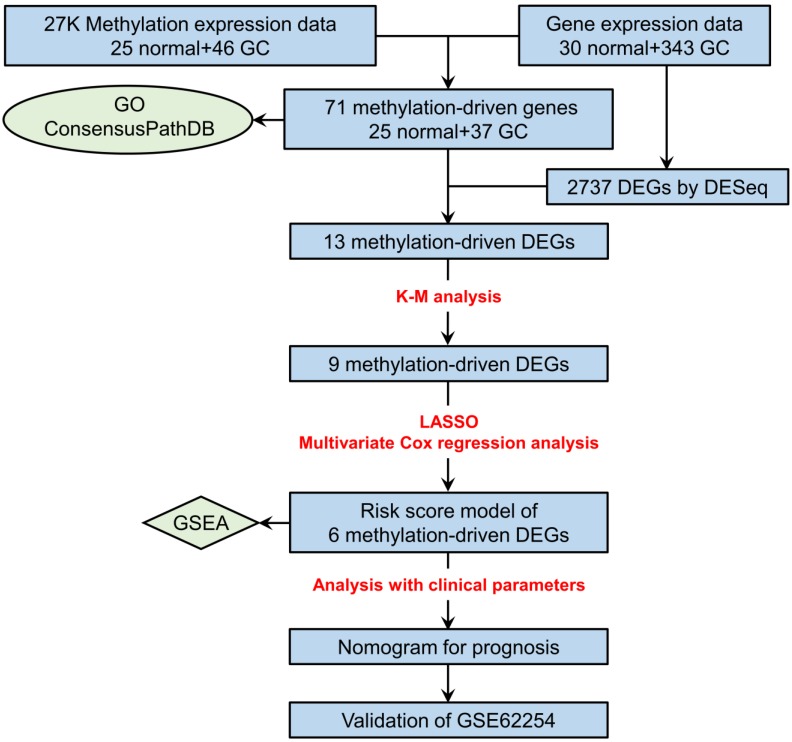
Flowchart depicting how prognostic genes were identified.

The study flowchart describing the process is shown in Figure 1. After the comparison of mRNA expression between GC tissues (n = 343) and adjacent nontumorous gastric tissues (n = 30), 2737 DEGs (|logFC| > 1, adjusted *P* value < 0.05) remained for further analysis. Among these genes, 649 DEGs were upregulated, and 2088 DEGs were downregulated (Table S1).

### Identification of DNAm-Driven Genes in GC

**Figure 2:**
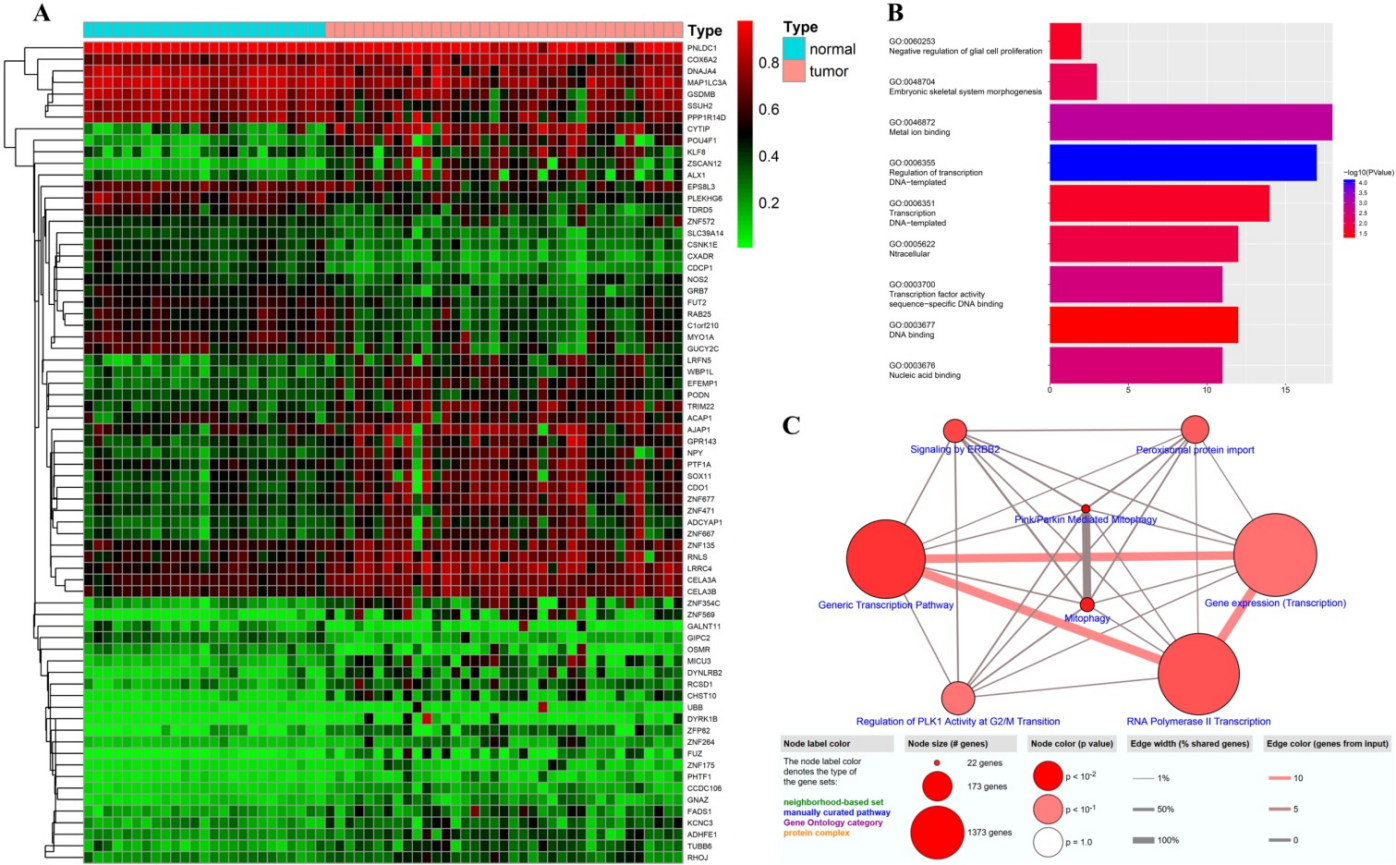
Candidate DNAm-driven genes screened by the Wilcoxon test. (A) Heatmap of the candidate DNAm-driven genes (n=71) in GC and nontumorous gastric tissues. (B) GO analysis of seventy-one DNAm-driven genes. (C) Pathway analysis based on multiple databases.

To identify DNAm-driven genes in GC, we performed MethylMix analysis^19^ on data from seventy-one clinical samples (Illumina Human Methylation 27 platform) downloaded from the TCGA. A total of seventy-one DNAm-driven genes (forty-seven hypermethylated and twenty-four hypomethylated) with an adjusted *P* value < 0.05 between the hyper- and hypomethylation groups and a correlation between DNAm and gene expression less than -0.3 were screened, and their methylation levels were visualized via a heatmap (Figure 2A, Table S2). GO analyses were performed with the aim of elucidating the functional characteristics of the identified DNAm-driven genes, and we obtained nine GO terms (*P* < 0.05; Figure 2B; Table S3). We found that the GO functions of these DEGs were significantly enriched in the following categories: “regulation of transcription, DNA-templated”, “metal ion binding”, “transcription factor activity, sequence-specific DNA binding”, “nucleic acid binding”, and “embryonic skeletal system morphogenesis” (*P* < 0.001). However, the Reactome database pathway analysis from ConsensusPathDB showed that the genes were enriched in Pink/parkin mediated mitophagy and mitophagy (*P* < 0.001) and six other pathways, namely, generic transcription pathway, signaling by ERBB2, RNA polymerase II transcription, peroxisomal protein import, gene expression (transcription), and regulation of PLK1 activity at G2/M transition (*P* < 0.05; Figure 2C; Table S4).

### Generation of a Prognostic Risk Score Model for GC

**Figure 3:**
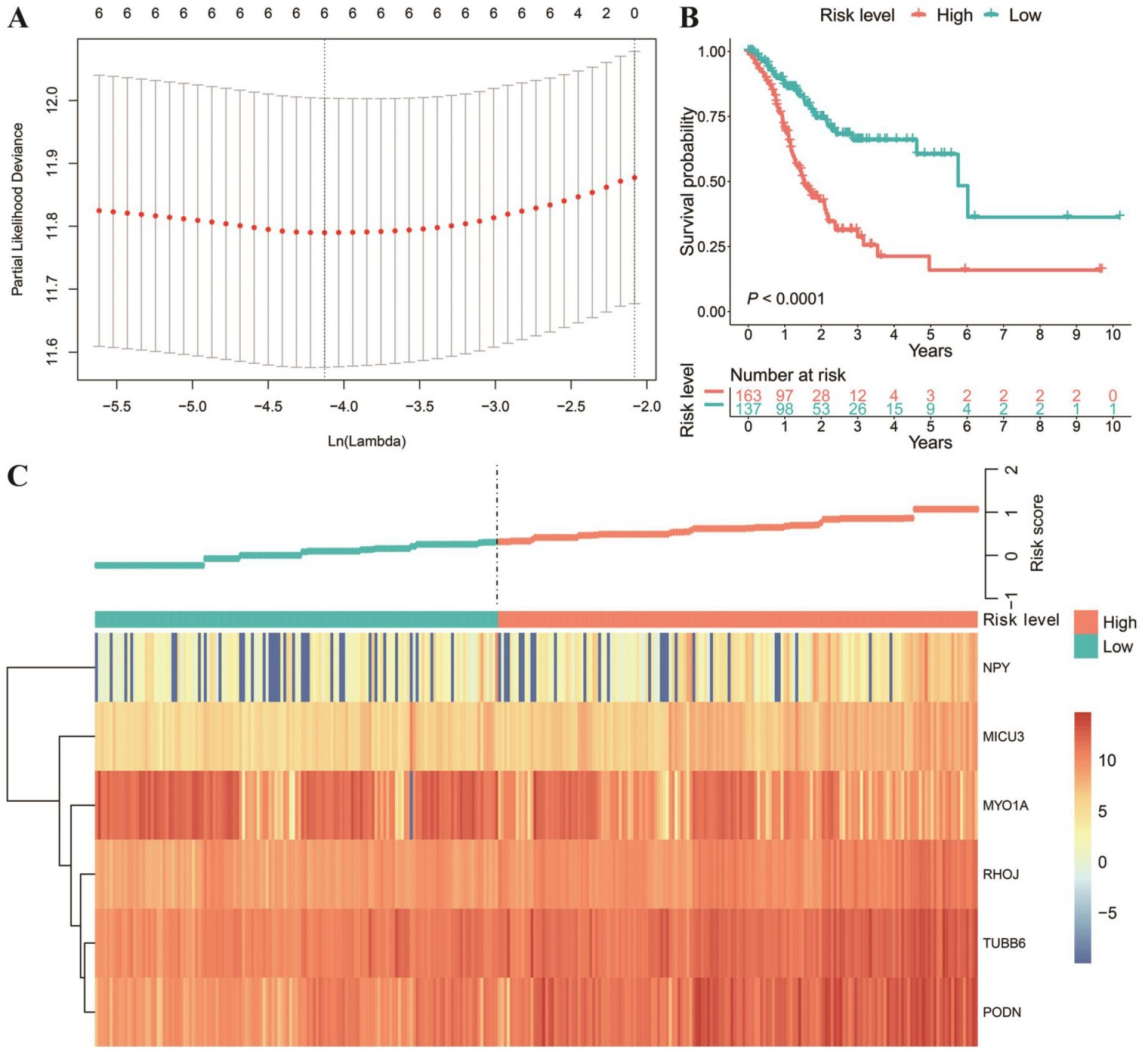
Texture feature selection and six-gene risk score model construction in the TCGA cohort. (A) Tuning parameter (λ) selection in the LASSO model used ten-fold cross-validation via the maximum criteria. The dotted vertical lines were drawn at the optimal values using the maximum criteria and the one standard error of the maximum criteria (the 1-SE criteria). (B) Comparison of OS between the high-risk score and low-risk score groups. (C) Heatmap of the six-gene expression profiles and distribution of corresponding risk scores in the high-risk and low-risk subgroups in the TCGA database.

Next, we intersected the DEGs and DNAm-driven genes. Then, we explored the relationship between the gene expression of thirteen DNAm-driven DEGs and OS by utilizing K-M analysis (Table S5). Of note, the X-tile approach was used to determine the optimal cut-off value. Among the thirteen included DNAm-driven DEGs, nine met the criteria for statistical significance via the log-rank test (*P* < 0.05) (Figure S1). The nine selected candidate DNAm-driven DEGs featured coefficients (not zero) in a further LASSO logistic regression model in which the selected genes were required to appear 1000 times of 1000 repetitions (Figure 3A). Finally, six DNAm-driven DEGs (*PODN*, *MYO1A*, *NPY*, *MICU3*, *TUBB6* and *RHOJ*) were selected as prognostic genes and presented in the risk score calculation formula. The predictive model was established by adding the product of the expression level and relative coefficient of each gene in the LASSO regression as follows: risk score = (0.2159037 * *NPY* mRNA level) + (0.2069438* *MICU3* mRNA level) + (-0.2337186 * *MYO1A* mRNA level) + (0.1574830 * *RHOJ* mRNA level) + (0.1584843 * *TUBB6* mRNA level) + (0.3310443 * *PODN* mRNA level). Positive coefficients of *PODN*, *NPY*, *MICU3*, *TUBB6* and *RHOJ* in the LASSO regression implied that their high expression represented poor OS in GC patients. Nevertheless, a high value of *MYO1A* indicated prolonged OS in both the training and validation datasets (Figure S1; Figure S2). For the 300 patients with full clinical data, we calculated the risk score based on the six-gene expression signature and identified the most compatible cut-off value with X‐tile diagrams. Those with a risk score over the cut-off value, 163 patients in total, were classified as the high-risk group, while the remaining 137 patients were classified as the low-risk group. The K-M analysis of the two groups showed that the OS of the high-risk score group was significantly shorter (*P* < 0.0001; Figure 3B). The gene expression profiles of all patients and the correlated risk scores were visualized as a heatmap (Figure 3C).

### Development and Evaluation of a Nomogram for OS Prediction in GC

**Figure 4:**
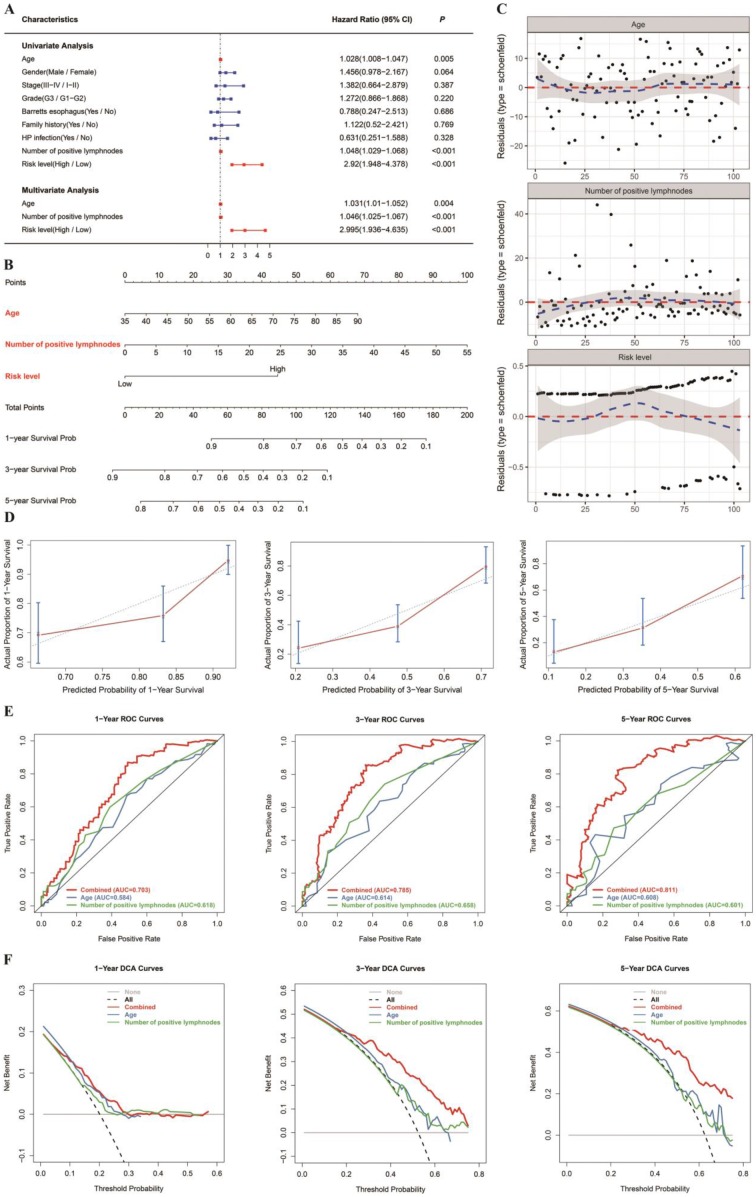
Nomogram to predict 1-, 3- and 5-year OS. The OS nomogram was developed in the TCGA cohort, with age, number of positive lymph nodes and risk level (DNAm signature) incorporated. (A) Univariate and multivariate analyses of the risk level, clinical factors and pathological characteristics with OS. The statistical significance level is indicated by different colors; red indicates statistical significance, and blue indicates no significance. (B) Nomogram to predict the 1-, 3- and 5-year OS of GC patients. (C) The Schoenfeld residual suggested that this model met the equally proportional risk hypothesis. Schoenfeld model residuals vs age, number of positive lymph nodes and risk level were plotted to obtain a preliminary assessment of which of these predictive factors should be incorporated into the model. (D) Calibration curves of 1‐, 3‐ and 5‐year OS. Blue dotted lines represent the ideal predictive model, and the red solid line represents the observed model. (E) Time‐dependent ROC analysis was used to evaluate the accuracy of the OS nomograms. The red, blue and green solid lines represent the combined model, age, and number of positive lymph nodes, respectively. (F) DCA curves evaluate OS nomograms from the perspective of clinical benefit and scope of clinical benefits. The y-axis represents the net benefit. The x-axis represents the predicted OS probability. The black dotted line represents the condition that all patients survive in 5 years, while the gray solid line represents the condition that none of the patients survive for more than one year. In the current study, the decision curve showed more benefit with a threshold probability > 0.0% using the OS nomogram.

Age, quantity of positive lymph nodes and risk level were regarded as significant predictive factors in univariate and multivariate regression analyses (Figure 4A). Taking all the above significant predictive factors into consideration, we generated a comprehensive nomogram (Figure 4B). In addition, Schoenfeld model residuals vs age, number of positive lymph nodes and risk level were plotted to obtain a preliminary assessment of whether these predictive factors should be incorporated into the model. Schoenfeld residuals suggested that this model met the equally proportional risk hypothesis (Figure 4C). The C-index and the robust C-index were 0.701 and 0.695, respectively. The calibration curve of the model for the possibility of OS at 3 years and 5 years demonstrated accurate predictive ability (Figure 4D). In addition, the prognostic capacity of the six-gene signature was demonstrated by the area under the curve (AUC) of the time-dependent ROC curve. Compared with age and number of positive lymph nodes, the AUC of the nomogram was increased (Figure 4E), indicating that the acuity of the nomogram was predominantly better than that of age or number of positive lymph nodes. DCA was performed to assess the clinical meaning. As demonstrated by the favorable probability, the combined model showed better net benefit than the age or number of positive lymph nodes only model, which indicates that the nomogram can help clinicians make more accurate assessment of patient prognosis. (Figure 4F). Because the nomogram was built based on more than one prognostic factor, it works better than each single factor alone. The model performed well in both the short- and long-term OS prediction, therefore, we have confidence in its potential to assist doctors in making medical decisions and GC patients in planning their follow-up schedules.

### External Validation of the Nomogram

**Figure 5:**
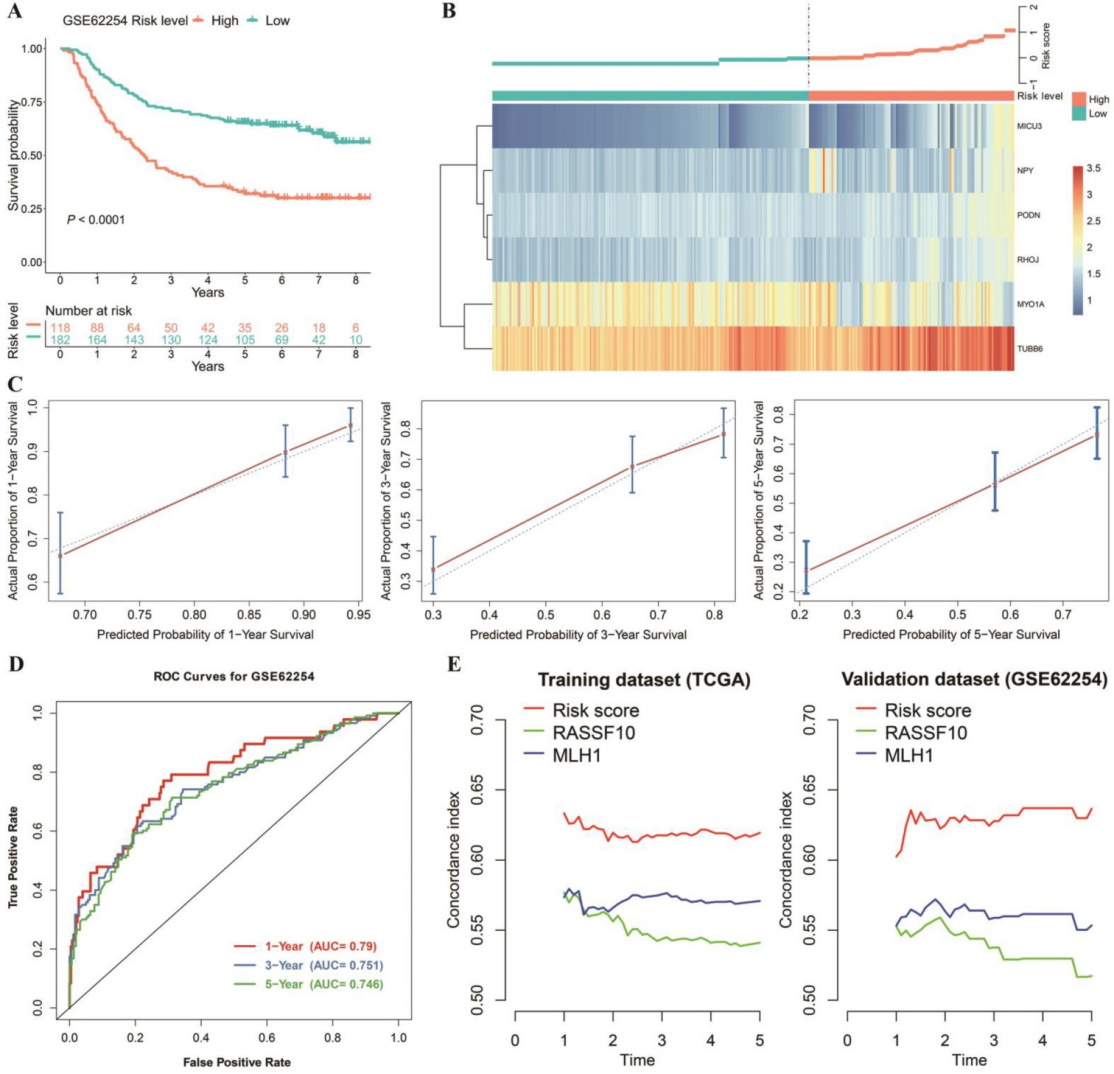
Validation of the prediction model. (A) OS was significantly lower in the high‑risk score group than in the low‑risk score group. (B) Heatmap and distribution of the six gene expression profiles in the high-risk and low-risk subgroups in the GEO database. (C) Calibration curve for the risk score model in the validation cohort. The blue dotted line represents the ideal predictive model, and the blue solid line represents the observed model. (D) ROC of the survival prediction model with the combined model, age, and number of positive lymph nodes in the validation dataset. (E) Concordance index of the indicated prognostic model in the training and validation datasets.

The nomogram mentioned previously was further validated in the GEO dataset GSE62254 (Figure 5A, B, C and D). The nomogram calibration curves for the possibility of 1-, 3- and 5-year OS displayed obvious concordance between the predicted results and observations in the GEO cohort (Figure 5C). Similar to the performance in the TCGA cohort (Figure 4E), the AUCs were 0.79, 0.751, and 0.746 for 1-, 3- and 5‐year survival times, respectively, in our validation dataset (Figure 5D). In addition, we compared the existing DNAm-driven gene prognostic models^23^, ^24^ with our risk score model based on the C-index from the first year to the fifth year. The results show that the risk score model performs best in both the training and the validation sets (Figure 5E).

### Methylation Degree and Gene Expression of the Six DNAm-driven DEGs in Cancer and Normal Samples

**Figure 6:**
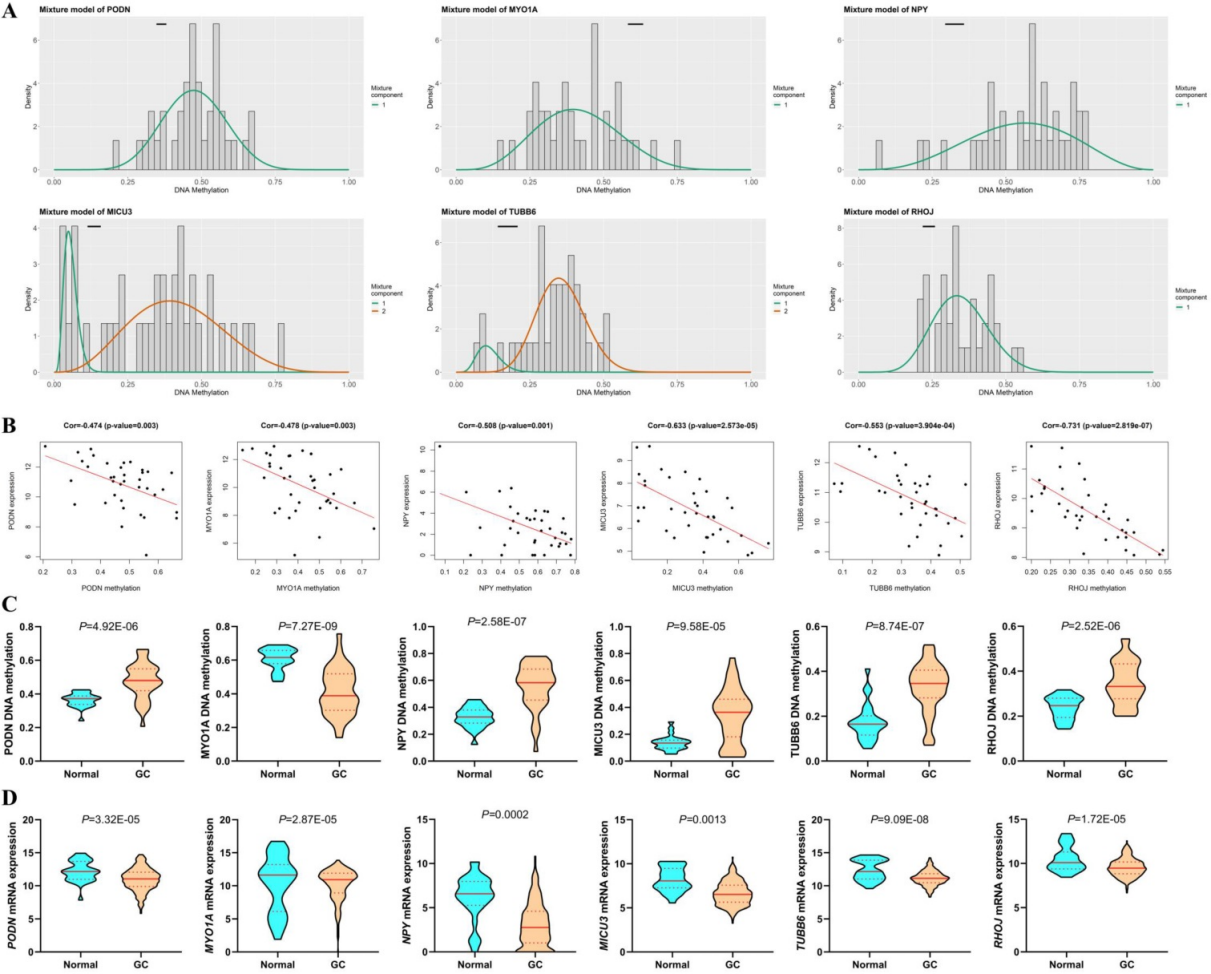
DNAm-driven genes. **(**A) Differential methylation statuses. The histogram demonstrates the distribution of *PODN*, *MYO1A*, *NPY*, *MICU3*, *TUBB6* and *RHOJ* methylation in GC samples. Beta values represent the methylation level (range from 0 to 1), and the horizontal black bar indicates the distribution of methylation values in the nontumorous gastric samples. (B) Regression analysis between the mRNA level and DNAm level of the six DNAm-driven DEGs. The vertical axis and the horizontal axis denote the mRNA level and DNAm level, respectively. (C) DNA methylation of the six DNAm-driven DEGs. (D) mRNA expression of the six DNAm-driven DEGs.

Among the six DNAm-driven DEGs, five (*PODN*, *NPY*, *MICU3*, *TUBB6* and *RHOJ*) were hypermethylated, while *MYO1A* was hypomethylated (Figure 6A and C) based on the TCGA GC cohort. As shown in Figure 6B, there is a significant inverse correlation between methylation and mRNA levels (|R|>0.3, *P* < 0.05). Moreover, the mRNA expression of hypermethylation-driven DEGs was decreased significantly in GC tissues compared with that in adjacent nontumorous gastric tissues (*P* < 0.05, Figure 6D).

### CNV, Mutation Characteristics and Kyoto Encyclopedia of Genes and Genomes (KEGG) Enrichment

**Figure 7:**
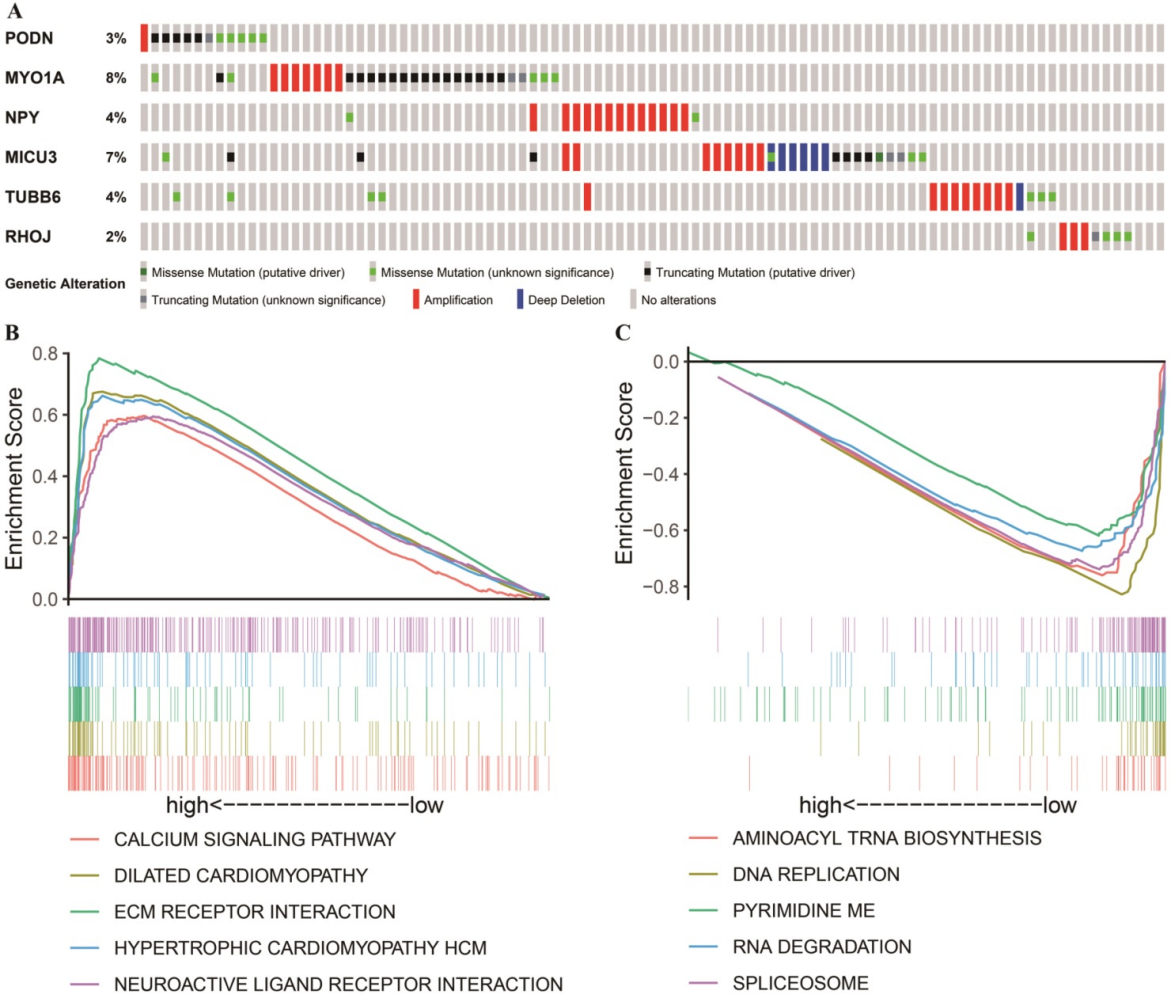
Genetic alterations and mutation characteristics of DNAm-driven DEGs and GSEA. (A) Genetic alterations of DNAm-driven DEGs in GC samples. The rows and columns indicate the genes and tumor samples, respectively. (B, C) Enrichment plots of the top five KEGG pathways in the high-risk score (Figure 7B) and low-risk score (Figure 7C) groups in GC.

Apart from being affected by methylation, the selected DNAm-driven DEGs (*PODN*, *MYO1A*, *NPY*, *MICU3*, *TUBB6* and *RHOJ*) are also affected concurrently by gene amplification, deletion and mutations (Figure 7A). By utilizing the GDC TCGA Stomach Cancer (STAD) database, we observed that the genetic alteration percentages of six genes ranged from 2%-8%, which had little contribution to mRNA expression. For example, there was no correlation between CNV and the mRNA expression of each gene after regression analysis (Figure S3). When the existing alterations that increase mRNA levels were ignored, the five hypermethylated genes still exhibited a relatively downward trend of mRNA levels compared to their expression in adjacent nontumorous gastric tissues. This finding indicates that DNAm plays a more critical role in GC in this study. However, the role of these alterations in determining the *MYO1A* mRNA level is difficult to estimate at present, and a high proportion of putative truncating mutations may attenuate its gene expression. To further characterize the potential signaling pathways involved in the influences on the risk score model, GSEA was performed to enrich the KEGG pathways in the two groups. A false discovery rate (FDR) less than 0.05 and an absolute value of the enrichment score (ES) greater than 0.5 were defined as the cutoff criteria. As shown in Figure 7B, the top five signaling pathways in the high-risk score group (risk score > 0.314) were “CALCIUM SIGNALING PATHWAY”, “DILATED CARDIOMYOPATHY”, “ECM RECEPTOR INTERACTION”, “HYPERTROPHIC CARDIOMYOPATHY HCM” and “NEUROACTIVE LIGAND RECEPTOR INTERACTION” while the top five signaling pathways in the low-risk score group (risk score < 0.314, Figure 7C) were “AMINOACYL TRNA BIOSYNTHESIS”, “DNA REPLICATION”, “PYRIMIDINE METABOLISM”, “RNA DEGRADATION” and “SPLICEOSOME”. The vast majority of the above signaling pathways are reported to be involved in tumor progression, laying the foundation for further exploring the molecular mechanisms of GC.

## Discussion

The lack of specific and sensitive biomarkers for predicting prognosis remains an urgent problem to be solved in the management of GC patients. Some prognostic models for GC patients have been reported. Recently, an ISGC classifier based on the ImmunoScore (IS) signature 25 was proposed to effectively predict patients with GC who would benefit from adjuvant chemotherapy. In tumor cells, alterations in the genome and epigenome can always be detected and have proven to be associated with certain tumor characteristics, such as oncogenic transformation and cellular proliferation 26. Considering that genome methylation is highly specific, herein, we first developed and validated a prognostic risk score model based on the DNAm signature and then combined this model with age and number of positive lymph nodes to construct an OS nomogram for predicting the prognosis of individual patients with GC. Of note, this model also has the potential to be widely applied after external validation and performed better than similar models reported previously 23, 24.

Aberrant methylation changes occur frequently in tumors. Among these deregulated DNAm-driven genes, some may promote malignant transformation via the overexpression of oncogenes or the knockdown of TSGs, which make up a new balance in the tumor microenvironment and have the potential to be predictive biomarkers for prognosis. With the advance of methylation sequencing, epigenetic changes are easy to identify with high sequencing depth and accuracy. Therefore, we utilized a model-based instrument (MethylMix)^19^ to identify DNAm-driven genes with aberrant methylation and linked these data to RNA-seq data that reflected gene expression. This integrative analysis has been performed in another cancer type^27^. It is worth mentioning that in our study, seventy-one preliminarily screened DNAm-driven genes were mainly enriched in gene expression-related signaling pathways, such as “RNA polymerase II transcription”, “generic transcription pathway”, “gene expression (transcription)”, and “regulation of PLK1 activity at G2/M transition”, which suggests that methylation changes in GC regulate gene expression. In our risk model, the expression of five genes (*PODN*, *NPY*, *MICU3*, *TUBB6* and *RHOJ*) was decreased in tumor tissues, and the greater the degree of downregulation was, the better the prognosis was, indicating that the hypermethylation of these genes may play a protective role in GC patients. In detail, the downregulation of DNAm-driven genes is a compensatory response to protect the organism. A high degree of downregulation is achieved through hypermethylation; if downregulation is insufficient under this condition, the stronger the protection will be, and the worse the prognosis will be. *MYO1A* may be a typical TSG because its hypomethylation always predicts a good prognosis. It is easy to understand that the downregulation of methylation levels in oncogenes and the upregulation of methylation levels in TSGs contribute to tumorigenesis. To clarify their potential mechanisms in affecting OS, GSEA was conducted to identify the relevant KEGG pathways in the high-risk and low-risk groups. Risk factors triggering the six dysregulated genes are enriched in several pathways, such as “ECM RECEPTOR INTERACTION”, “NEUROACTIVE LIGAND RECEPTOR INTERACTION” and “HYPERTROPHIC CARDIOMYOPATHY HCM”. Previous studies have found that some genes enriched in “dilated cardiomyopathy” and “hypertrophic cardiomyopathy HCM” signaling pathways closely related to multiple cancer types. For instance, TGFβ1 stimulates THBS1 expression in oral squamous cell carcinoma (OSCC) cells. THBS1 promotes the expression of matrix metalloproteinases (MMPs) partly through integrin signaling, thereby favoring OSCC invasion 28. Another gene, TGFβ3, directly induces the upregulation of stromal POSTN expression. Hence, the growth, migration and invasion of head and neck cancer cells are accelerated 29. A comparative genomic analysis of oral versus laryngeal and pharyngeal cancer also found that LAMA2 (TCGA: 5% vs 19%) mutations are enriched in laryngeal and pharyngeal squamous cell carcinoma (L/P-SCC)^30^, while other factors are enriched in pathways such as “DNA REPLICATION” and “PYRIMIDINE METABOLISM”. Overexpression of the key metabolite cytidine related gene ENTPD8, which is enriched in the “PYRIMIDINE METABOLISM” pathway, was reported to promote cell apoptosis and inhibit proliferation by promoting CTP metabolization into cytidine in pancreatic cancer tissue (PCT)^31^.

Among the six DNAm-driven DEGs, podocan, a protein of the small leucine-rich proteoglycan (SLRP) family encoded by the *PODN* gene, was found to be a potent regulator of the cellular phenotype in the extracellular matrix (ECM). ECM molecules are highly effective modulators of cell functions, such as migration and proliferation 32. Given the inhibitory effect of high podocan levels on smooth muscle cell (SMC) proliferation 33, PODN may also be involved in cell proliferation regulation, which requires further experimental validation. Ras homolog family member J (RHOJ), a member of the Rho GTPase family, acts as a molecular switch by regulating cell functions, such as migration and proliferation, correlating well with increased cell motility and invasiveness 34. This finding is consistent with our results, and the specific regulatory mechanism by which this gene impacts GC is still unknown. As a sympathetic neurotransmitter highly relevant to tumor biology, neuropeptide Y (NPY) is released from activated peripheral sympathetic neurons under chronic stress or hypoxia. The release of NPY can regulate many bioprocesses (e.g., stimulate cell proliferation, migration and survival, and regulate cell differentiation) 35. Aberrant *NPY* methylation is involved in tumorigenesis 36. Mitochondrial calcium uptake family member 3 (MICU3), a paralog of MICU1, which likely arose by gene duplication and exhibits high expression levels in the brain, encodes an EF-hand-containing protein that functions by interacting with MICU1, forming a dimer and enhancing MCU-dependent mitochondrial Ca^2+^ uptake 37. Mitochondrial Ca^2+^ regulates various cellular events, including tumorigenesis. Abnormal fatty acids, such as cis-9, 10-methyl-octadecanoic acid (MOA), caused by *Helicobacter pylori* (HP) infection, serve as activators of protein kinase C (PKC) in a Ca^2+^-dependent manner. Interestingly, PKC has been implicated in regulating the proliferation activity of gastric epithelial cells and the malignant transformation process, associated with the increased proliferation of gastric epithelial cells and linked with GC 38. In this study, the *MICU3* gene showed a hypermethylated state and relatively low expression in GC, which may disturb the mitochondrial Ca^2+^ uptake function, thus playing a role in regulating the cellular and molecular functions of GC cells. Tubulin beta 6 class V (*TUBB6*) was recognized as a potential mutation hot spot in human colorectal cancers accompanied by microsatellite instability 39 and serves as a biomarker for predicting GC peritoneal metastasis 40. Brush border protein myosin Ia (MYO1A), which plays an essential role in polarization and differentiation in colon cancer, is highly expressed in normal gastric epithelial cells, suppressing intestinal tumors. In colorectal tumors, epigenetic regulation often inactivates its expression. Despite relatively sparse CpG islands, promoter methylation has been observed in several colon cancer cell lines and primary colorectal tumors 41. Existing studies have also shown that MYO1A can suppress tumorous changes in the normal gastric epithelium, indicating that MYO1A may serve as an important protective factor 42. Methylation was negatively correlated with *MYO1A* mRNA expression in our study, and MYO1A plays an inhibitory role in the progression of GC. In addition, *MYO1A* mRNA levels were simultaneously affected by an 8% mutation rate, which may contribute to nonsense expression in GC. Indeed, *MYO1A* is among the most frequently mutated genes in some types of GC.

To the best of our knowledge, the six-gene predictive model has not been previously published, and it will help to identify new prognostic biomarkers in GC from a clinical perspective. Moreover, our signature based on specific genes is easy to test routinely, considering its cost-effectiveness. There are also some shortcomings to this study. To detect GC outcomes, it was recently proposed to increase the amount of research on clinical biomarkers, such as epiregulin 43, the albumin-to-globulin ratio (AGR) and the lymphocyte-to-monocyte ratio (LMR) 44 in patients with GC. DNAm has expanded the field of cancer research, attracting an increasing number of scientists. However, although a favorable performance in external validation indicates its potential, it is too early to conclude that our two-dimensional model (epigenetic and transcriptional signatures) is preferable to traditional examinations, such as medical imaging evaluation, in directly predicting patient outcomes. Therefore, further experimental verification is required. In addition, although the nomogram incorporates age, number of positive lymph nodes and risk level to successfully predict the OS of GC patients, the clinical characteristics were considered insufficient due to limited information in the study cohorts. In the future, it will be necessary to construct a better prognostic nomogram derived from more centers with complete clinical information and sequencing data.

In summary, a risk score prediction model comprising six DNAm-driven DEGs was identified and validated, and this model combined with other clinical factors could produce a good prognostic nomogram for GC patients. Our findings support the assumption that genes tightly controlled by DNAm are likely related to tumor outcomes. Importantly, only six genes were used to build the prognostic model. Measuring the expression levels of these six DNAm-driven genes can provide a cost-effective and accurate prediction for the prognosis of GC in clinical practice.

In conclusion, our study established a nomogram that combined the DNAm signature, age and number of positive lymph nodes and is cost effective in clinical practice, advancing the individualized prediction of OS in GC patients with high sensitivity and specificity.

## Supplementary Material

Supplementary figures and table legends.

Supplementary table 1.

Supplementary table 2.

Supplementary table 3.

Supplementary table 4.

Supplementary table 5.

## Abbreviations

AGR: albumin-to-globulin ratio
C-index: Harrell's concordance index
CNV: copy number variation
DAVID: Database for Annotation, Visualization and Integrated Discovery
DCA: decision curve analysis
DEG: differentially expressed gene
DIRC1: disrupted in renal cancer 1
DNAm: DNA methylation
DNMT3A: De novo methyltransferase
ECM: extracellular matrix
ES: enrichment score
FDR: false discovery rate
FGFR: fibroblast growth factor receptor
GC: gastric cancer
GDC: Genomic Data Commons
GEO: Gene Expression Omnibus
GSEA: Gene Set Enrichment Analysis
HP: Helicobacter pylori
IS: ImmunoScore
K-M: Kaplan-Meier
LASSO: least absolute shrinkage and selector operation
LMR: lymphocyte-to-monocyte ratio
MICU3: mitochondrial calcium uptake family member 3
MMP: metalloproteinase
MOA: methyl-octadecanoic acid
MYO1A: myosin Ia
NPY: neuropeptide Y
OS: overall survival
OSCC: oral squamous cell carcinoma
PCT: pancreatic cancer tissue
PKC: protein kinase C
PTX: paclitaxel
RHOJ: Ras homolog family member J
ROC: receiver operating characteristic
SLRP: small leucine-rich proteoglycan
SMC: smooth muscle cell
TCGA: The Cancer Genome Atlas
TSG: tumor suppressor gene
TUBB6: Tubulin beta 6
XCI: X-chromosome inactivation
