# Supplementary material for: Development and Validation of a Prognostic Nomogram for Gastric Cancer Based on DNA Methylation-Driven Differentially Expressed Genes
Source: Int J Biol Sci. 2020 Feb 10;16(7):1153–65. doi: 10.7150/ijbs.41587 (PMC7053317; doi:10.7150/ijbs.41587)
Supplement: Supplementary file 1 — Supplementary figures and table legends. [file ijbsv16p1153s1.pdf]

Supplementary materials

Figure S1

K-M analysis of the nine candidate DNAm-driven DEGs in the TCGA database.

Figure S2

K-M analysis of the six DNAm-driven DEGs in the GEO validation database.

Figure S3

Regression analysis between CNV and gene expression of the six DNAm-driven DEGs.

Table S1

DEGs identified by DESeq.

Table S2

Methyl Mix models for the DNAm-driven genes.

Table S3

Results of the GO analysis.

Table S4

Pathway analyses based on multiple databases.

Table S5

K-M analysis of the thirteen candidate DNAm-driven DEGs.

Figure S1

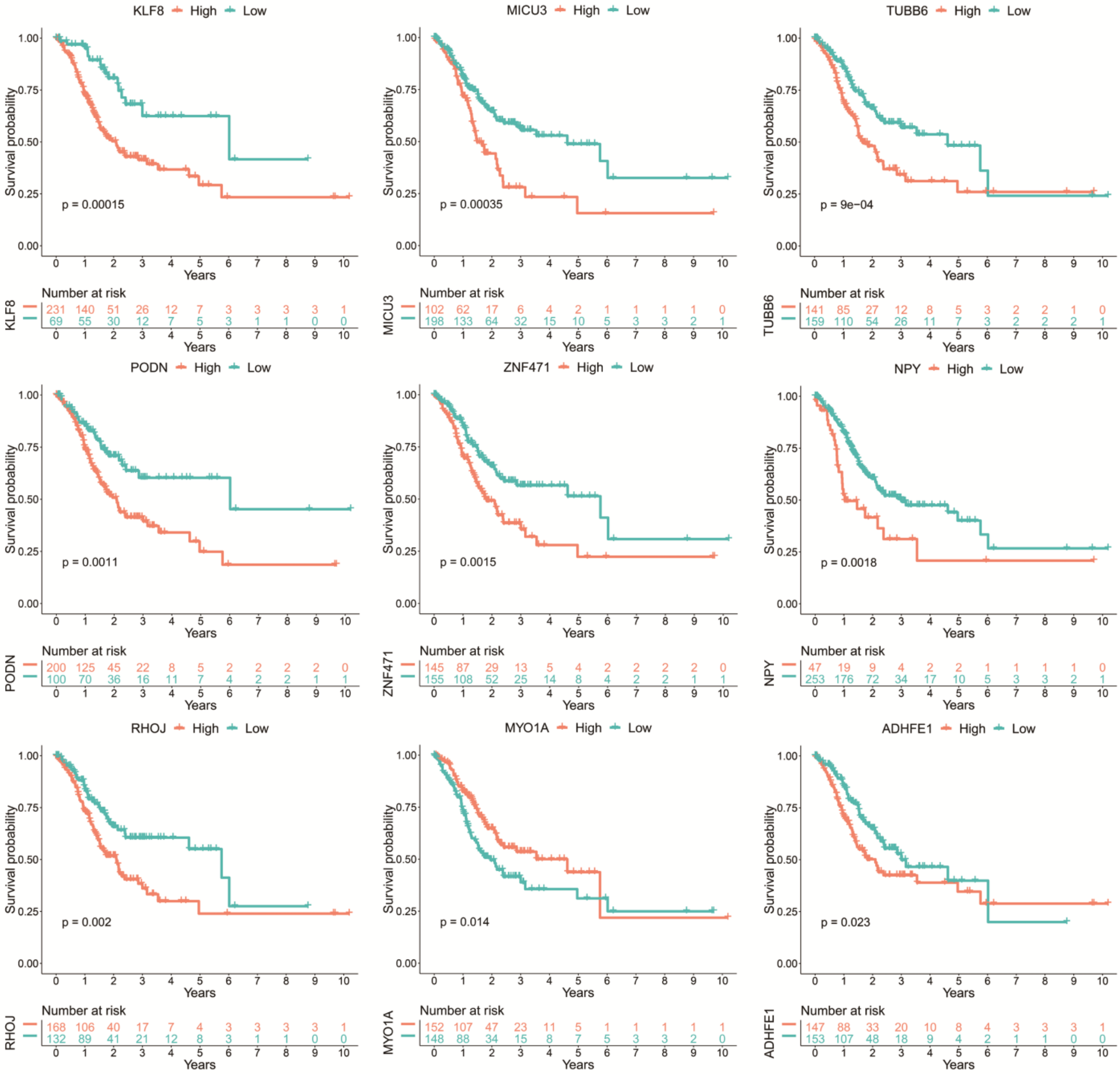

KLF8 High Low
Survival probability
1.00
0.75
0.50
0.25
0.00
p = 0.00015
0 1 2 3 4 5 6 7 8 9 10
Years
Number at risk
KLF8
231 140 51 26 12 7 3 3 3 3 1
69 55 30 12 7 5 3 1 1 0 0
MICU3 High Low
p = 0.00035
MICU3
102 62 17 6 4 2 1 1 1 1 0
198 133 64 32 15 10 5 3 3 2 1
TUBB6 High Low
p = 9e−04
TUBB6
141 85 27 12 8 5 3 2 2 1 0
159 110 54 26 11 7 3 2 2 2 1
PODN High Low
p = 0.0011
PODN
200 125 45 22 8 5 2 2 2 2 0
100 70 36 16 11 7 4 2 2 1 1
ZNF471 High Low
p = 0.0015
ZNF471
145 87 29 13 5 4 2 2 2 2 0
155 108 52 25 14 8 4 2 2 1 1
NPY High Low
p = 0.0018
NPY
47 19 9 4 2 2 1 1 1 1 0
253 176 72 34 17 10 5 3 3 2 1
RHOJ High Low
p = 0.002
RHOJ
168 106 40 17 7 4 3 3 3 3 1
132 89 41 21 12 8 3 1 1 0 0
MYO1A High Low
p = 0.014
MYO1A
152 107 47 23 11 5 1 1 1 1 1
148 88 34 15 8 7 5 3 3 2 0
ADHFE1 High Low
p = 0.023
ADHFE1
147 88 33 20 10 8 4 3 3 3 1
153 107 48 18 9 4 2 1 1 0 0

Figure S2

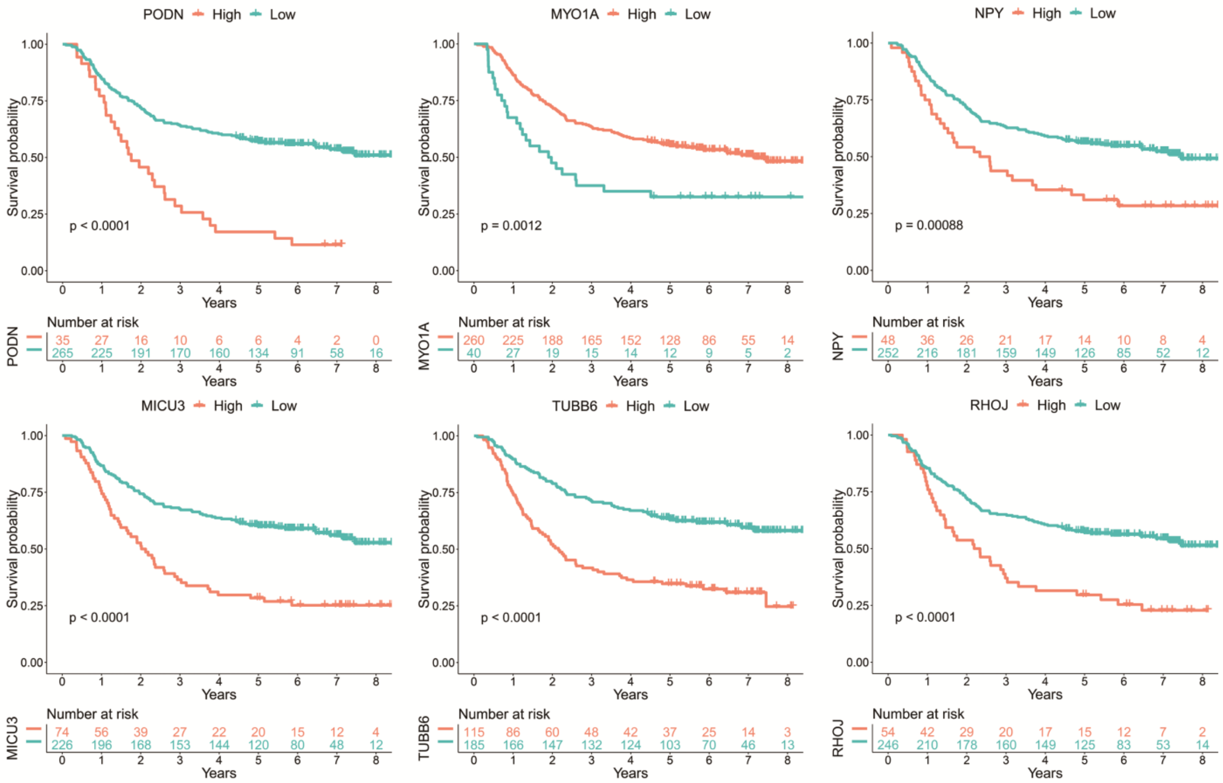
PODN High Low
p < 0.0001
MYO1A High Low
p = 0.0012
NPY High Low
p = 0.00088
MICU3 High Low
p < 0.0001
TUBB6 High Low
p < 0.0001
RHOJ High Low
p < 0.0001
Survival probability
Years
Number at risk

Figure S3

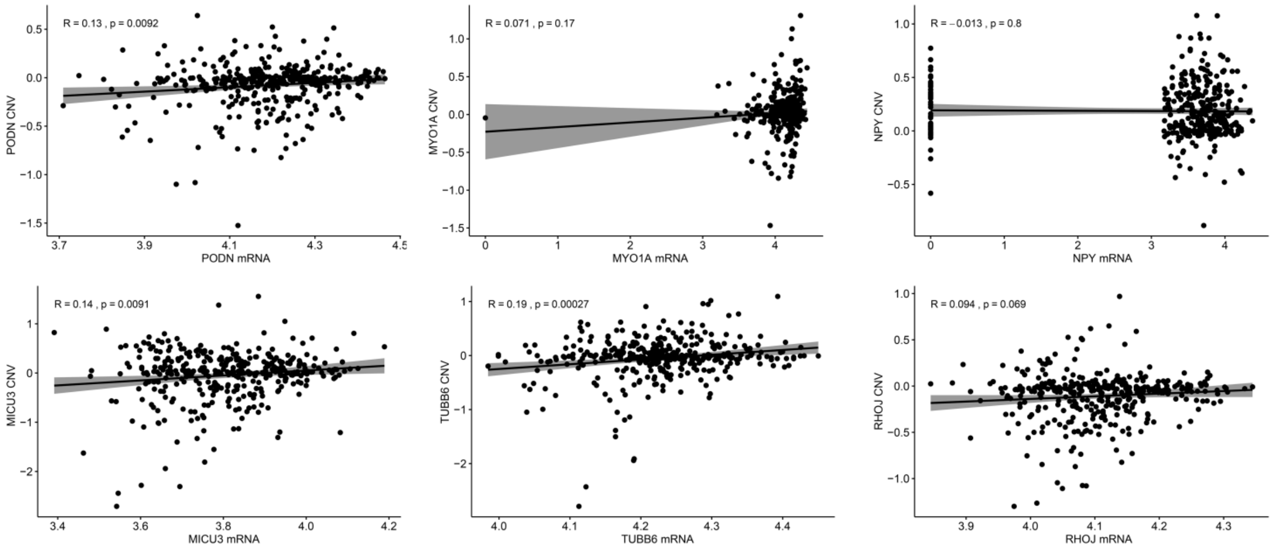
R = 0.13 , p = 0.0092
PODN CNV
PODN mRNA
R = 0.071 , p = 0.17
MYO1A CNV
MYO1A mRNA
R = −0.013 , p = 0.8
NPY CNV
NPY mRNA
R = 0.14 , p = 0.0091
MICU3 CNV
MICU3 mRNA
R = 0.19 , p = 0.00027
TUBB6 CNV
TUBB6 mRNA
R = 0.094 , p = 0.069
RHOJ CNV
RHOJ mRNA
